# Tracking the Dynamics of Mind Wandering: Insights from Pupillometry

**DOI:** 10.5334/joc.41

**Published:** 2018-07-19

**Authors:** Claudia Pelagatti, Paola Binda, Manila Vannucci

**Affiliations:** 1 Department of NEUROFARBA-Section of Psychology, University of Florence, IT; 2 Department of Translational Research and New Technologies in Medicine and Surgery, University of Pisa, IT; 3 Institute of Neuroscience, CNR, Pisa, IT

**Keywords:** mind wandering, pupillometry, spontaneous mind wandering, task-unrelated thoughts, task disengagement

## Abstract

Mind wandering (MW) refers to the shift of attention away from a primary task towards thoughts unrelated to the task. Here we show that significant new insight into the dynamics of this process can be gained by using pupillometry. Participants performed a monotonous vigilance task which was intermixed with task-irrelevant verbal cues. At fixed times, we interrupted them and asked what they were thinking about immediately prior to the probe and what had triggered their thought. We observed pupil dilation after the presentation of verbal cues reported to have triggered MW, compared with other verbal cues with similar emotional content. Thus, MW is associated with pupil dilation. We also analysed the pupil-constriction response to the task-stimuli (vertical and horizontal bars, to be categorized as targets and non-targets for the vigilance task), and found that this was unchanged during MW. We conclude that pupil size provides an index of MW, objective and covert and that this may be exploited in further studies to understand whether and how MW affects the processing of sensory stimuli.

## 1. Introduction

In our daily lives, we frequently find our attention drifting away from an ongoing task, focusing on unrelated private thoughts and feelings such as memories or prospective thoughts. In the majority of cases, this *“shift in the focus of attention away from the here and now towards one’s private thoughts and feelings”* (Smallwood, O’Connor, Sudbery, Obonsawin, 2007, p. 818) occurs spontaneously. We refer to this as mind wandering (MW; for a review: Smallwood & Schooler, 2015).

Experience-sampling studies have shown that humans spend between 25% and 50% of their waking hours engaged in MW (see for a review, Smallwood & Schooler, 2015). The experience of MW is associated with both costs and benefits. For example, higher rates of MW during lectures at the university are associated with lower comprehension and retrieval for the lecture material (e.g., Risko, Anderson, Sarwal, Engelhardt, & Kingstone, 2012). In other contexts, MW can lead to even more serious consequences, such as traffic accidents (e.g., studies on MW in drivers and professional pilots) and medical malpractice (e.g., Smallwood, Mrazek, & Schooler, 2011). Nevertheless, MW can also be adaptive since it promotes future planning and simulation, personal problem solving and decision making, as well as self-reflection and the maintenance of a sense of self-identity and continuity across time (Smallwood & Schooler, 2015). Given the frequency of MW in daily life and its complex psychological functions, there is much interest in revealing the cognitive and neural mechanisms that underlie this phenomenon.

In most laboratory studies, MW is investigated by asking participants to perform a monotonous, undemanding or over-practiced task and intermittently sampling their experience using thought-probes. These probes ask participants to provide self-reports about the contents of their experience (e.g., whether their attention was on-task or off-task/MW immediately before the probe). Research on MW has greatly benefited from the adoption of the “strategy of triangulation” (Smallwood & Schooler, 2015), whereby self-reports at probes, behavioural measures (i.e., task performance) and physiological measures (e.g., fMRI, ERPs, eye-movements, pupillometry) are combined, and used to make inferences about covert mental experiences.

One key question is what triggers MW episodes. In the MW literature, MW episodes have been mainly described as self-generated (e.g., Smallwood, 2013) and stimulus-independent (Antrobus, 1968), emphasizing their independence from external stimuli and ongoing actions. However, it is theoretically possible that external stimuli act as triggers for MW episodes (see also Klinger, 2013 for a discussion of this issue). For example, reading the word “broken glass” might trigger the retrieval of an autobiographic memory: our mind might wander to that day when we stepped on a broken glass hidden in the sand of the beach, while we were on vacation. This theoretical possibility is verified in a number of recent studies, showing that environmental stimuli indeed trigger MW episodes (Maillet & Schacter, 2016b; Maillet, Seli, & Schacter, 2017; McVay & Kane, 2013; Plimpton, Patel, & Kvavilashvili, 2015; Song & Wang, 2012; Vannucci, Pelagatti, & Marchetti, 2017). For example, in a study by Plimpton et al. (2015), participants were engaged in a monotonous vigilance task while being simultaneously exposed to irrelevant cue-words (i.e., ‘relaxing on a beach’ or ‘crossing the street’). Participants were stopped 11 times during the vigilance task and recorded their thoughts at that moment (thought-probes). The results revealed that the majority of task-unrelated thoughts had an identifiable external trigger, and of these, 85% were reported by participants as having been triggered by the cue-words. Consistent findings come from Vannucci et al. (2017), who compared the number of MW episodes between two groups of participants who performed a monotonous task with and without intercalating verbal cues. Compared to the No-cues group, the Verbal-cues group reported a higher number of MW episodes, which were mostly triggered by the irrelevant cue-words.

Capitalizing on the MW triggering effect of task-irrelevant cue-words, here we employ a paradigm similar to the one used by Plimpton et al. (2015) and aim to track the dynamics of MW by means of a physiological measure, pupil diameter.

A significant body of literature has shown that the diameter of our eye pupils is affected by the cognitive and emotional demands of the task. For example, the pupils dilate in response to increased cognitive processing load (Hess & Polt, 1960; Kahneman & Beatty, 1966) and emotional load (Partala & Surakka, 2003). In addition, recent work has shown that the amplitude of pupillary response to light stimuli is indicative of the level of attention paid to those stimuli: the response is stronger when the light is attended (Binda & Murray, 2015; Binda, Pereverzeva, & Murray, 2013; Mathôt, van der Linden, Grainger, & Vitu, 2013; Naber, Alvarez, & Nakayama, 2013), indicating that pupil diameter is a rich source of information about the dynamics of cognition and perception.

Previous pupillometry studies of MW have yielded contradictory results. In some studies, off-task reports (presumably reflecting MW) were associated with larger pupil diameter compared to on-task reports (e.g., Franklin, Broadway, Mrazek, Smallwood, & Schooler, 2013), and larger pupil diameters characterized participants who retrospectively reported more MW compared to those who reported less MW (Smallwood et al., 2012). This may be explained by higher cognitive and emotional load during MW. However, other studies found the reverse pattern (e.g., Grandchamp, Braboszcz, & Delorme, 2014; Mittner et al., 2014; Unsworth & Robison, 2016). For example, in a recent study, Unsworth and Robison (2016) examined changes in pupil diameter associated with on-task and different lapses of attention, specifically, MW, inattentive states/non alert (i.e., blank mind) and external distractions experienced during a challenging attention-control task. The authors found that when participants reported being externally distracted, their baseline pupil diameters were much larger than when they were on-task, and when participants reported either MW or being inattentive, their baseline pupil diameter was much smaller than when on-task.

The authors interpreted these findings in line with the “landscape of attentional lapses” proposed by Lenartowicz, Simpson, and Cohen (2013), according to which lapses of attention are determined by the combination of arousal level and the direction of attention (whether it is directed to external stimuli or to internal thoughts). Unsworth and Robison (2016) associated MW experienced during the challenging and demanding task to arousal decrease, fatigue, and reduced alertness.

In the present study, we use pupillometry to track the dynamics of MW triggered by verbal cues. To this end, we compare the time course (over 6 seconds) of pupil diameter observed in 3 conditions: after a verbal cue that triggers MW (MW trigger), after a verbal cue with emotional content which is followed by an on-task period, and after any other control verbal cue. As in previous studies we also compare changes in pupil diameter associated with MW and on-task reports at the probes. We distinguish MW from external distraction, and we further differentiate between intentional and spontaneous MW. Several studies have shown that MW and external distractions are partially distinct and that they can be differentiated at the behavioural (Stawarczyk, Majerus, Catale, & D’Argembeau, 2014; Unsworth & McMillan, 2014; Vannucci et al., 2017) and physiological level (e.g., Unsworth & Robison, 2016). A growing number of studies have also shown that spontaneous and deliberate forms of MW are dissociable cognitive experiences (see for a review, Seli et al., 2016).

In addition, our pupillometry approach allows for addressing a second question, whether MW affects one’s ability to respond to sensory events. Multiple lines of evidence point to a reduction of sensory responses during MW. For example, Smallwood et al. (2011) showed reduced evoked response to task-relevant stimuli during MW periods. Similarly, Kam et al. (2011) found that sensory-evoked responses to task-irrelevant visual stimuli measured by ERPs were significantly attenuated during off-task compared to on-task states. Here we measure pupillary responses to weak light stimulation (the stimulus for the undemanding over-practiced task that forms the background of our MW paradigm) and ask whether these pupillary light responses are reduced during MW.

With these measures, we aim to strengthen the evidence that pupillometry provides quantitative and reliable information on the internal state of a participant. This, together with standard behavioural measures and other physiological techniques, contributes to defining the occurrence and time course of individual MW episodes and, therefore, ultimately to understanding the underlying processes.

## 2. Method

### 2.1. Participants

Fifty undergraduate students from the University of Florence (41 females, age range 18–27, *M* = 20.84 years) volunteered to participate in the study. All participants were Italian native speakers, and they had normal or corrected-to-normal vision. The experimental protocol is consistent with the declaration of Helsinki and with the regulations of the University of Florence that hosted the study.

### 2.2. Apparatus

Subjects sat in front of a monitor screen, with their heads stabilized by chin rest. Viewing was binocular. Stimuli were generated with the PsychoPhysics Toolbox routines (45, 46) for MATLAB (MATLAB r2010a, The MathWorks) and presented on a LCD colour monitor (Asus MX239H, 51 × 28 cm placed at 57 cm viewing distance) with a resolution of 1920 × 1080 pixels and a refresh rate of 60 Hz, driven by a Macbook Pro Retina (OS X Yosemite, 10.10.5). All stimuli were shown in white (55 cd/m^2^) against a black background (0.05 cd/m^2^). Two-dimensional eye position and pupil diameter were monitored binocularly with a CRS LiveTrack system (Cambridge Research Systems) at 30 Hz, using an infrared camera mounted below the screen. Pupil diameter measures were transformed from pixels to millimeters after calibrating the tracker with an artificial 4 mm pupil, positioned at the approximate location of the subjects’ left eye. Gaze position data were linearized using a standard 9-point calibration, run prior to each session.

### 2.3. Vigilance task

**Figure 1:**
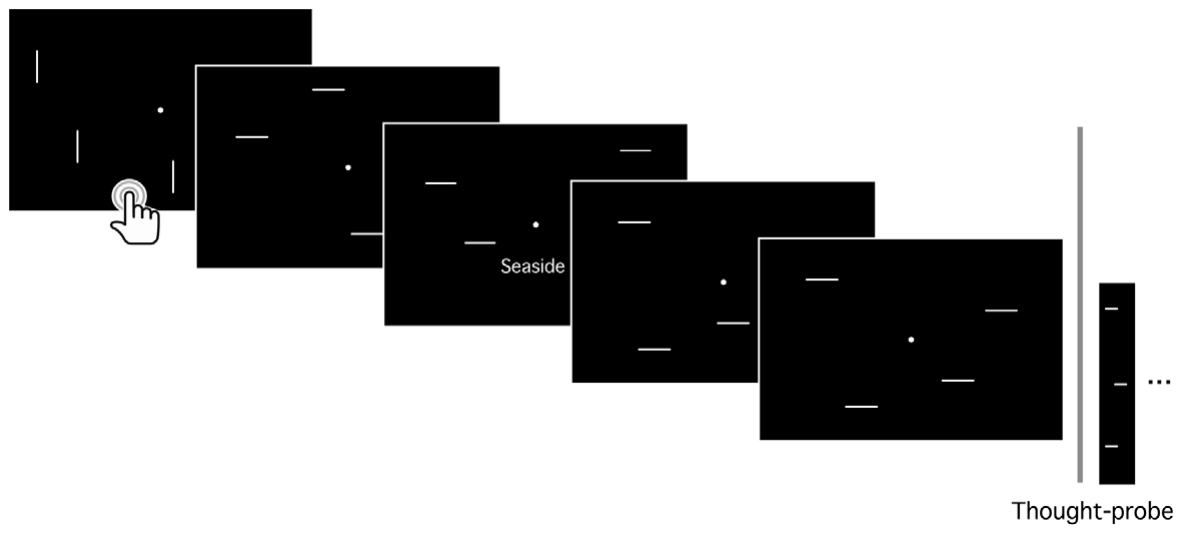
Experimental paradigm: vigilance task with task-irrelevant cue-words. A thought-probe method was used to collect self-reports about the subjects’ focus of attention during the task. Participants were asked to focus on the central fixation point and press the spacebar whenever a target (vertical bars) was detected. At 28 fixed points, the task was stopped by a thought-probe, which included the following questions: (i) “What were you thinking about just immediately prior to the probe?”; if participants reported a thought they were asked to (ii) give orally a short description of their mental content, (iii) indicate if the thought occurred spontaneously, if they deliberately decided to think about it or if they were not sure about the answer, and (iv) whether the thought had been triggered by the environment, by their own thoughts, by a word on the screen (if so, to specify the word) or if there was no trigger (see Method section).

Participants completed a modified version of the computer-based vigilance task developed by Schlagman and Kvavilashvili (2008) and used in previous studies (e.g., Plimpton, Patel & Kvavilashvili, 2015; Vannucci, Pelagatti, Hanczakowski, Mazzoni, & Rossi Paccani, 2015; Vannucci, Pelagatti, & Marchetti, 2017). The task consisted of 1120 trials, presented in a fixed order, each lasting 2 s. A white fixation point (0.2 deg diameter) was presented in the centre of the screen for each trial. In each trial, an image was shown depicting a pattern of white horizontal (non-target stimuli) or vertical lines (target stimuli) (4.1 × 0.2 deg) randomly distributed across the screen, against a black background (see Figure 1). Target stimuli appeared on 68 trials, and they were presented pseudo-randomly, every 9–31 trials; participants were asked to press the space bar whenever a target was detected. Moreover, a white verbal cue (e.g., “long hair”, “exquisite dinner”; 0.88 deg height) was placed under the fixation spot, in 210 trials (see Figure 1). The verbal cues were selected from the Italian adaptation of a standardized pool of 800 word-phrases developed by Schlagman and Kvavilashvili (2008) and used in previous studies (for more details on the adaptation, see Vannucci et al., 2015). Equal numbers of neutral (*n* = 70), positive (*n* = 70), and negative (*n* = 70) cues were presented during the task.

In order to collect participants’ state (on-task, MW, or distracted in other ways), the vigilance task was stopped at 28 fixed points (separated by an average of 40 trials, *SD* = 7.50, corresponding to an average of 80 sec, *SD* = 15 sec), and a thought-probe appeared on the screen. Each thought-probe included the same questions: (i) “What were you thinking about just immediately prior to the probe?”, response options: “I was focused on the task” (on-task, OT), “I was thinking about something” and “My mind was blank”; if participants reported a thought they were asked to (ii) give orally a short description of their mental content and (iii) indicate if the thought occurred spontaneously (i.e., simply popped into their mind), if they deliberately decided to think about it or if they were not sure about the answer, and (iv) whether the thought had been triggered by the environment, by their own thoughts, by a word on the screen (if so, to specify the word), or if there was no trigger.

Sessions started with 20 practice trials, and a 5-minutes break was allowed between the two halves of the task, for a total session duration of approximately 120 min. Please see appendix A for more details on the verbal instructions given to the participants.

### 2.4. Analysis

Analyses were carried out on 42 out of 50 participants.^1^ Before conducting the analyses, all reports given by participants to thought-probes were classified into distinct categories (as in Vannucci, Pelagatti, & Marchetti, 2017, and Unsworth & Robison, 2016; for a similar categorization, see also Stawarczyk et al., 2011, 2014). The categories were (i) on-task reports: when participants reported that their attention was fully focused on the task; (ii) external distractions reports: any thought whose content was about current sensory perceptions unrelated to the task, either exteroceptive perceptions (i.e., a noise outside the room) or interoceptive sensations (i.e., bodily sensations); (iii) task-related reports: any thought related to some task features or to the participant’s overall performance (i.e., thoughts about the experiment’s duration); (iv) MW reports: any thought whose content was unrelated to the task at hand and attention was decoupled from the current environment (v) blank mind reports: when participants reported that their attention was not focused on the task and they appeared not thinking about anything at all, their mind was a complete blank.

An off-line analysis examined the eye-tracking output to exclude time-points with unrealistic pupil-size recordings (values outside the 90^th^ percentile of each 2s long trial) and then interpolated the remaining time-points at 20 Hz. This procedure yielded smooth and consistent pupil traces, excluding only 4.45% of trials due to excessive signal loss (>60% of the time-points). Note, however, that this approach retains for analysis trials where a blink might have occurred.

In a first analysis, we compared the time course of pupil diameter observed in 3 conditions: after a verbal cue later reported by the participant as the trigger of a spontaneous MW episode (MW trigger), after a verbal cue with emotional content which was followed by on-task period, and after any other control verbal cue (i.e., all the cue-words that were neither MW triggers nor emotional cue-words followed or preceded by on-task periods). Trials were sorted based on their timing relative to a cue later identified as triggering or non-triggering a MW event (0, 1, 2 trials after the word). We used as “baseline” pupil diameter the average diameter at the reference event (trial “0” where the word was presented). We studied the time course of pupil diameter over trials after subtracting this baseline.

In a second analysis, we compared pupil diameter relative to a MW report versus an OT report or any other response (1, 2, 3 trials before the thought-probe that interrogated the participant on his/her thoughts). We used as “baseline” pupil diameter the average diameter at the reference event (the trial coded as “1”, in Figure 3, is the trial immediately preceding the probe, i.e., the last trial before the probe).

In a third analysis, we evaluated the response to the light bars in trials immediately preceding a MW/OT report; in this case, the baseline pupil diameter in the first 250 ms of each individual trial was subtracted from the trace, allowing for evaluation of the amplitude of the light evoked pupillary constriction.

Statistical analyses utilized a linear-mixed model approach, motivated by the considerable sample size variability across subjects. In this approach, individual trials from all subjects are compared with a model comprising both the effect of experimental variables (“fixed effects”) and the variability across participants (“random effects”). Random effects were coded by allowing subject-by-subject variations of the intercept of the model. In all cases, the dependent variable is “baseline corrected pupil diameter”, which we obtained by averaging pupil diameter in a pre-specified temporal window of each trial (e.g., in the interval 500: 1000, when the pupil-constriction in response to the task-stimulus is expected to peak), and subtracting the average pupil diameter in a reference temporal window (e.g., in the first 250 ms of the trial); please refer to the results section for specific definitions of the temporal windows for averaging and baseline-subtractions. We used standard MATLAB functions provided with the Statistics and Machine Learning Toolbox (R2015b, The MathWorks). Specifically, the function “fitlme (data, model)” fit the linear-mixed model to the data, yielding an object “lme” with associated method “ANOVA” that returns *F* statistics with associated degrees of freedom, and *P* values for each of the fixed effect terms and “CoefTest” for post-hoc comparisons.

## 3. Results

Performance on the vigilance task was near-perfect for all participants; out of 68 targets, there were 0.33 (*SD* = 0.69) misses and 0.79 (*SD* = 1.14) false alarms. Upon task interruption, participants gave a total of 309 of on-task (OT) reports (option “I was focused on the task”) (*M* = 7.36, *SD* = 5.52, range 0–19) and 503 MW reports (*M* = 11.98, *SD* = 5.02, range 2–22). Out of the all MW, 402 were reported as spontaneous MW (*M* = 9.57, *SD* = 4.26, range 2–19) and 88 were reported as intentional MW episodes (*M* = 2.10, *SD* = 2.13, range 0–8). Given the low amount of intentional MW episodes, these reports were not further considered in our analyses and we focused only on spontaneous MW.

Of the 402 spontaneous MW episodes, 212 were reported by participants as triggered by a specific word-phrase cue shown on screen (*M* = 5.05, *SD* = 2.59, range 1–12). There was a mean of 8.97 trials, *SD* = 10.37 (*M* = 17.94 sec., *SD* = 20.74 sec.), between a MW trigger and the thought-probe where the report of MW triggered by that specific cue was given.

**Figure 2:**
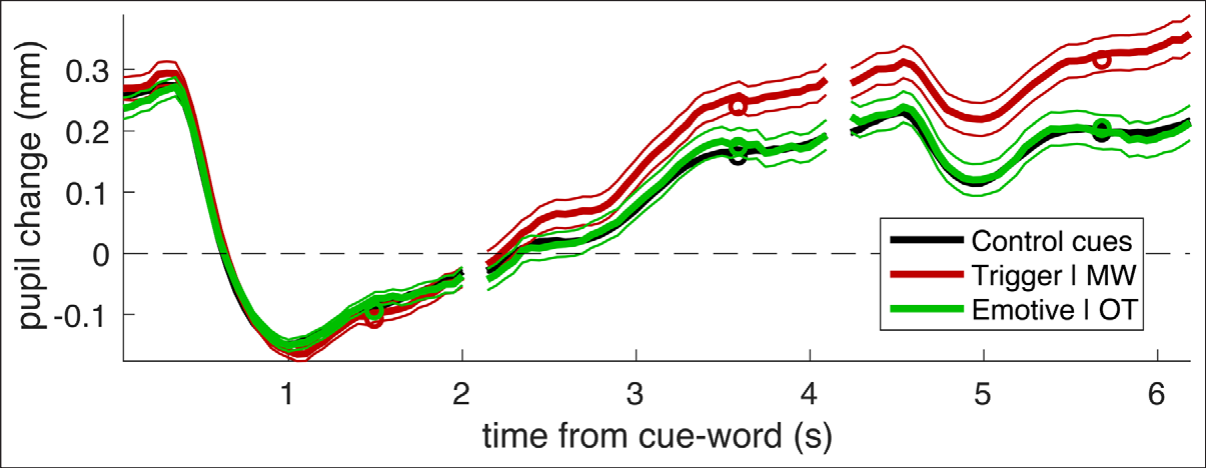
Pupil traces aligned to the average pupil diameter during the presentation of the cue-word. Thick lines give the average across all trials, thin lines show the s.e. and circles show the average values entered the LMM analysis (average over the second half of each trial).

In the first analysis of pupillometry data, we analysed the time course of pupil diameter over two trials after the cue later identified as triggering or non-triggering a MW event. Figure 2 shows the traces, aligned to the average pupil diameter during the word presentation. As expected, words (white text on black background) evoked a strong pupillary constriction: the light response, which recovered over several seconds. However, pupil diameter increased more when the cue-word triggered MW (MW trigger) compared to other cues with emotional content which were followed by on-task (non-trigger) periods, as well as compared to other “control” cue-words (i.e., all the cue-words that were neither MW triggers nor emotional cue-words followed or preceded by on-task periods).

For this analysis, we only considered those words that were followed by at least two trials with successful pupil recording. This yielded a 6-sec time course of pupil diameter, from which we subtracted the average pupil diameter during the first 2-sec interval (the first trial with the cue-word presentation). This left 194 MW triggers and 215 emotional words followed by an OT report (non-trigger), plus 7391 of “control” cue-words. For the statistical analysis of the traces, we summarized time courses by taking the average pupil diameter in the last second of each trial (the farthest from the reference).

These values were entered in a Linear-Mixed Model analysis, with two fixed-factors: type of cue-word (MW trigger, emotional followed by OT, other control words) and time from the cue (coded as number of trials), plus the random effect of subjects modelled as a variable intercept of the model. This revealed a significant interaction between the two fixed factors (F(2,23394) = 10.21610, p = 0.00004), which we further analysed with a series of post-hoc tests. These showed that cue-type had a significant effect over the two trials that followed the cue-word, where there was a significant difference between pupil diameter following MW triggers and emotional words followed by an OT report (first trial following the cue-word: F(1,407) = 4.12330, p = 0.04295; second trial following the cue-word: F(1,407) = 8.51346, p = 0.00372); MW triggers were also significantly different from the other control cue-word (first trial after the cue-word: F(1,7583) = 12.66170, p = 0.00038; second trial: F(1,7583) = 19.97163, p = 0.00001), whereas in no case were emotional words followed by OT different from the other control cue-words (first trial: F(1,7604) = 1.93229, p = 0.16455; second trial: F(1,7604) = 2.23358, p = 0.13508). To obtain an indicator of the reliability of our analysis, we also re-ran this analysis twice, after splitting the trials in half (odd and even). In spite of the (factor of 2) reduction of sample size, the primary effect of an interaction between time and trial type remained strong and significant (odd trials: F(2,11691) = 4.04089, p = 0.01761; even trials: F(2,11697) = 7.05208, p = 0.00087).

This suggests that pupil diameter does change (increases) during MW, providing a reliable indicator of the increased mental load (cognitive and emotional processing) that is involved in MW – compared with a simple vigilance task.

**Figure 3:**
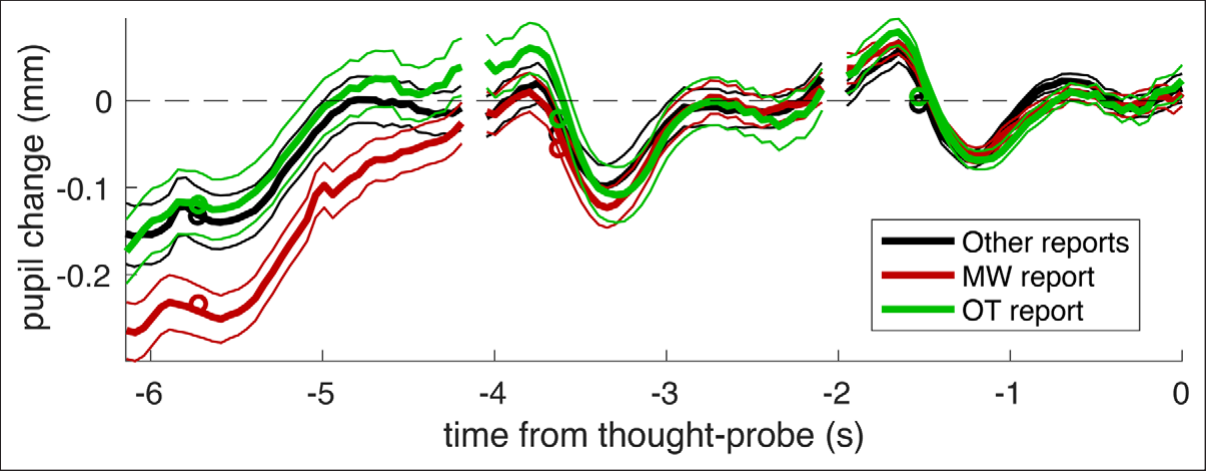
Pupil traces aligned to the average pupil diameter during the last trial before the thought-probe. Thick lines give the average across all trials, thin lines show the s.e. and circles show the average values entered the LMM analysis (average over the first half of each trial).

These findings were confirmed with the second analysis, aligning traces to the thought-probe (i.e., question about the attentional state) and separating those where reports of MW, OT or other reports were given (Figure 3). Also in this case, there was a relative pupil dilation preceding the MW report, which significantly differentiated MW trials from both other categories – whereas OT and other responses were not different.

To show this statistically, we aligned traces to the average pupil diameter on the last trial before the thought-probe, then assessed pupil diameter on each trial as the mean pupil diameter in the first second of the trial (the farthest from the reference). We only considered thought-probes that were preceded by at least three trials with successful pupil recording and subtracted the average pupil diameter during the last trial from the resulting 6-sec time course. This left 188 MW reports and 144 OT reports, plus 236 of “other” reports. The Linear-Mixed model analysis revealed a significant interaction between the fixed-factors type of report (MW/OT/others) and time from the thought-probe, coded as number of trials from the report: 3, 2 or 1 trial from the thought-probe (F(2,1698) = 3.89778, p = 0.02047). At three trials preceding the thought-probe, pupil diameter leading to a MW or OT reports could be clearly differentiated (F(1,330) = 6.71401, p = 0.00999). This confirms that there is more pupil dilation leading up to a MW report than there is to an OT/other report. Note that this cannot be explained by a confounding effect of foregoing cue-words being unevenly distributed before MW and OT reports. We verified this by showing that cue-words were equally likely to occur on the 4^th^ trial before a thought-probe (corresponding to times from –8 to –6 seconds in Figure 3) irrespectively of the subject report on the probe. The probability of a cue-word being shown on the 4^th^ trial before the thought-probe was: 75.37% +– 2.83% (mean and standard errors across participants) for probes with a MW report, 73.77% +– 4.36% for probes with a OT report and 75.05% +– 2.73% for probes with other reports.

**Figure 4:**
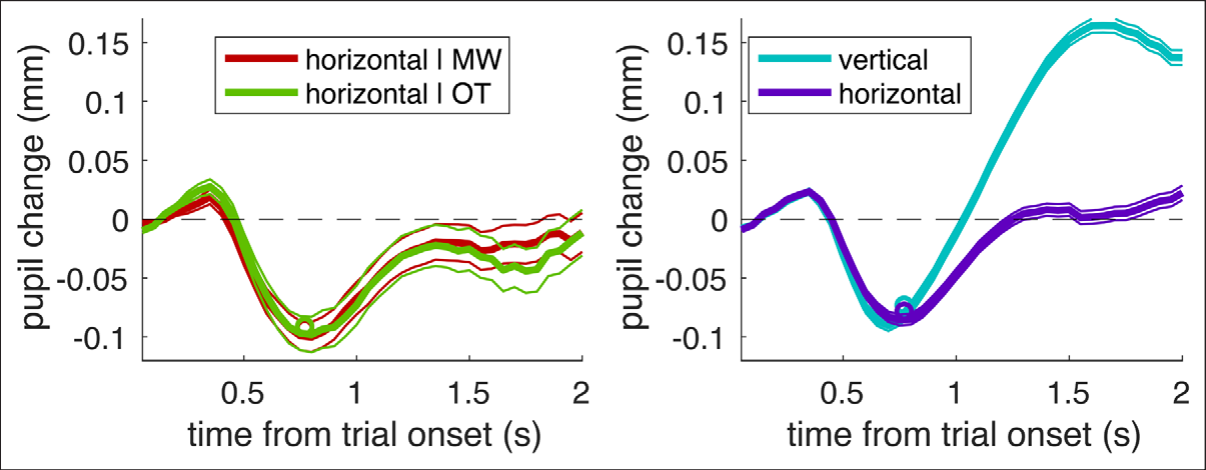
Pupil traces aligned to the average pupil diameter during the first 250 ms of each trial. Thick lines give the average across all trials, thin lines show the s.e. and circles show the average values entered the LMM analysis (average over the [.5:1]s interval). Left: trials with the horizontal bars that preceded a thought-probe at which MW or OT was reported. Right: trials with the frequent horizontal bars vs. the infrequent vertical bars that participants had to detect for their vigilance task.

To address our second question, whether sensitivity to sensory stimuli is weakened during MW, we turned to a different parameter of the pupil response. Rather than looking at the raw pupil diameter, we analysed the pupil constriction evoked by the bar stimuli (subtracting from each trial the baseline pupil diameter in the first 250 ms and then computing the average constriction in the interval [500:1000 ms] into the trial, where the pupil response peaks). If MW implies reduced sensitivity or attention to sensory stimuli, the pupil constriction in response to the bar stimuli should be reduced during MW compared to OT period. However, we did not find evidence in support for this prediction. We analysed the light response in the last trial before a thought-probe in which a MW or an OT report had been collected (including only trials that were not preceded by a cue-word that would shift the baseline and mask the light response, see Figure 2) and we did not find a significant difference between the two (F(1,645) = 0.02219, p = 0.88163). Figure 4 (leftmost panel) shows that light response had equal amplitude regardless of whether participants reported to be MW (372 usable traces) or OT (298 usable traces), suggesting that sensory and attentional processing of the simple stimuli used in the vigilance task is unaffected. We support this conclusion with a Bayesian analysis: computing a two-sample t-test between trials in the MW and OT conditions (t(644) = –0.08 p = 0.940), for which the Bayes Factor (BF)^2^ is 0.089. Conventionally, BF > 3 is strong evidence in favour of the experimental hypothesis (that the two samples are different) and BF < 1/3 is strong evidence in favour of the null hypothesis (that the two samples are not different). Thus, a BF of 0.089 strongly supports the conclusion that the pupil constriction response to the bar stimuli does not differ between the MW and OT trials.

We also analysed pupil constriction during the [500:1000 ms] interval associated with the two types of task-relevant trials: the frequent non-targets (horizontal bars) and the infrequent targets (vertical bars, see Figure 4 rightmost panel). This was because we expected these to be associated to marked differences in attentional processing: the infrequent targets being more attended than non-targets for both bottom-up factors (being infrequent) and top-down factors (being the stimuli that subjects had to signal by bar press). Thus, based on prior research that attended stimuli evoke a stronger pupil response (Binda & Murray, 2015), one might expect a stronger pupil constriction in response to the infrequent target trials. However, we did not find significant differences in the pupil response associated with the infrequent vertical bars (2735 usable traces) versus the frequent horizontal bars (only the last trial before a “target” included in this figure, amounting to 2520 usable traces; in both cases trials were never preceded by a cue-word; F(1,5059) = 0.00177, p = 0.96642). Also in this case, the Bayesian analysis supported the conclusion that there is no difference between the two samples of trials (two-sample t-test: t(5021) = 0.77 p = 0.442; BF = 0.043, clearly smaller than 1/3 and implying strong evidence in support of the null hypothesis).

## 4. Discussion

Multiple recent studies have investigated the contribution of the external environment to MW, consistently showing that MW might be triggered by external stimuli (Maillet et al., 2017; Maillet & Schacter, 2016b; McVay & Kane, 2013; Plimpton et al., 2015; Song & Wang, 2012; Vannucci et al., 2017). In the present study we capitalize on these findings and extend them further, by combining self-report measures with pupillometry. Our experimental paradigm is the same as in recent MW studies (Plimpton et al., 2015; Vannucci et al., 2017) and closely resembles the approach of several recent studies on spontaneous cognition (e.g., Barzykowski & Niedzwienska, 2016; Barzykowski & Staugaard, 2015; Cole & Berntsen, 2015; Cole, Staugaard, & Berntsen, 2016; Mazzoni, Vannucci, & Batool, 2014; Schlagman & Kvavilashvili, 2008; Kvavilashvili & Schlagman, 2011; Vannucci et al., 2015; Vannucci, Batool, Pelagatti, & Mazzoni, 2014; Vannucci, Pelagatti, Chiorri, & Mazzoni, 2016). Specifically, we asked participants to perform an undemanding vigilance task with task-irrelevant cue-words, that might act as trigger for MW. During the task, participants were presented with thought-probes and asked regarding the contents of their experience and the trigger of their thoughts, if any.

Tracking pupil size over periods of 6 seconds after a trigger and a non-trigger (i.e., a cue-word associated with an OT period; any other control cue-words) allowed us to monitor the dynamics of MW, tracking its unfolding over time.

We found a significantly larger pupil dilation following cue-words reported by participants as the trigger of MW compared to non-trigger words (which could have similar emotional content), and the pupil dilation appears to increase over time. This result suggests that a change in pupil diameter follows the onset of MW and it accompanies its unfolding and maintenance over time. We obtained the same pattern, comparing the pupil size recorded before the MW and OT probes.

Considering previous findings on the association between baseline pupil diameter and cognitive and emotional load (Hess & Polt, 1960; Kahneman & Beatty, 1966), the larger pupil diameter recorded after the onset of MW episodes can be explained in terms of the increased cognitive and emotional processing associated with MW, compared to the vigilance task (see for similar results, Franklin et al., 2013; Smallwood et al., 2012). In line with this interpretation, the contents of the MW episodes revealed the complexity of these thoughts, since most consisted of personal projections into the personal past (i.e., autobiographical memories referring to specific events) and the future (e.g., future planning, upcoming personal events), including emotional states and responses, rather than simple images or generic considerations.

In addition, we studied the pupil constriction evoked by the simple visual stimuli (bars) employed in the vigilance task, to test the hypothesis that the pupil response to sensory stimuli is reduced during MW periods. According to the perceptual decoupling hypothesis (Schooler et al., 2011), the processing of sensory input should be decreased when the mind wanders toward internal information and this perceptual decoupling would contribute to maintaining MW, by insulating the internal train of thought from the distracting influence of external information. However, in the present study, we did not find any significant differences between light responses to stimuli (bars) presented during MW and OT periods. Methodological differences between our study and previous studies finding an impairment of sensory processing during MW (e.g., Smallwood et al., 2011) might be responsible for this discrepancy. We recorded and analysed pupil responses to very simple visual stimuli, namely luminance bars, whereas Smallwood et al. (2011) employed more complex and meaningful visual stimuli – numbers. Profound differences exist in the effects of divided attention on simple visual features and complex stimuli, especially linguistic stimuli; only the former typically suffers no costs of divided attention, consistent with the hypothesis of unlimited-capacity parallel processing of multiple simple stimuli (Busey & Palmer, 2008; Palmer, 1994; White, Runeson, Palmer, Ernst, & Boynton, 2017). Consistent with these results, our findings suggest that the interference between different sources of information (i.e., external stimuli versus internally generated information, leading to the reduced sensory processing during MW), might selectively occur for perceptually and semantically complex stimuli. However, we must also consider the possibility that the attention modulation of pupil responses during MW is too weak to be detected in our set-up. Future studies are required to compare attentional modulations in MW and during more standard attentional modulations – for example, future studies might compare the effect of MW with the effect of cueing attention away from a sensory stimulus, which is known to reduce stimulus-evoked constriction (Binda & Murray, 2015).

Future studies are also needed to investigate whether and how the level of meta-awareness of MW might affect changes in baseline pupil diameter. Previous studies on MW have shown that individuals routinely fail (at least temporarily) to notice that their minds have wandered, as they are only intermittently aware of their internal state (see for a review, Schooler et al., 2011). This pattern has been revealed when participants were prompted by the experimenter to report the contents of their minds (probe-caught method) and asked how aware they were of where their attention was focused immediately prior to the probe. In response to this direct query, participants reported varying levels of meta-awareness, sometimes being fully aware that their mind had wandered, whereas at other times reporting their mind having wandered without being aware of it until the probe was presented. Interestingly, when participants classify their MW episodes as unaware, their performance (Smallwood, McSpadden, & Schooler, 2008) and neurocognitive activity (Christoff et al., 2009) systematically differ from when they report having been already aware that they were mind wandering. In the present study, we did not assess meta-awareness, and we could not distinguish between aware and unaware spontaneous MW episodes. However, in light of previous findings, one might argue that unaware MW, being more profound and disruptive to concurrent task performance, might be associated with a higher baseline pupil diameter compared to aware MW.

Finally, in the present study we investigated the experience of MW in a sample of young adults. Future studies are also needed to extend our investigation to other populations of special interest for research on MW, such as elderly people. Studies on aging have shown a reduction in MW in healthy older adults compared to young adults, and an age-related increase in reliance on the environment (see for a discussion, Maillet & Schacter, 2016a). Maillet and Schacter (2016b) found that, during an incidental-encoding task, older adults reported a reduction in proportion of thoughts cued by internal stimuli, but an increase in proportion of thoughts cued by external stimuli, compared to young adults. Combining our behavioural paradigm with pupillometry might advance our understanding of the neurophenomenology of the subjective experience of spontaneous MW in elderly and help clarifying the mechanisms underlying aging-related changes.

## Additional File

The additional file for this article can be found as follows:

10.5334/joc.41.s1 Appendix A. Vigilance Task instructions.

## Data Availability

Data and analyses scripts are available at osf.io/ywzec.

## Footnotes

Five participants were excluded for noncompliance with task instructions; two participants were excluded for self-reported mental illness and one for technical failures in data recording.

The Bayes Factor represents the likelihood ratio of the data given the null hypothesis and given the experimental hypothesis; values smaller than 1 indicate that the data are more likely given the null hypothesis than given the experimental one; values larger than 1 indicate that the data are more likely under the experimental hypothesis than under the null one. Conventionally, a threshold likelihood ratio of 3:1 (i.e. 3 or 0.333) is chosen to indicate significant evidence in support of either hypothesis.
