# Supplementary material for: Tracking the Dynamics of Mind Wandering: Insights from Pupillometry
Source: J Cogn. 2018 Jul 19;1(1):38. doi: 10.5334/joc.41 (PMC6634319; doi:10.5334/joc.41)
Supplement: Appendix A. — Vigilance Task instructions. [file joc-1-1-41-s1.pdf]

Appendix A. Vigilance Task instructions

Participants were asked to detect target stimuli (vertical lines) among a large number of non-target stimuli (horizontal lines), by pressing a space bar each time they detected a target stimulus. They were also told that they would see words in some of the trials but they were not supposed to do anything with these words. It was explained that the condition they were taking part in was looking at how people could keep their concentration on the patterns and that, in another condition, participants would have to concentrate on the words. Participants were informed that, due to the task being quite monotonous, they could find themselves thinking about other things (e.g., thoughts, plans, considerations, past events, images, etc.) and that these thoughts might simply pop into their mind spontaneously or they might be intentionally generated. They were told that they would be interrupted during the task and presented with thought-probes consisting of questions about their focus of attention just immediately prior to the probe.
